# Immune reconstitution inflammatory syndrome following treatment of cutaneous tuberculosis with rifampin, isoniazid, pyrazinamide, and ethambutol

**DOI:** 10.1016/j.jdcr.2023.12.004

**Published:** 2023-12-27

**Authors:** Ralina Karagenova, Bao Xin Liang, David J. Elpern, Dylan E. Lee, Casey M. Phan, Douglas W. Johnson

**Affiliations:** a John A. Burns School of Medicine, University of Hawaii at Manoa, Honolulu, Hawaii; b The Skin Clinic, Williamstown, Massachusetts; c Department of Dermatology, The Queen’s Medical Center, Honolulu, Hawaii; d Department of Pathology, The Queen’s Medical Center, Honolulu, Hawaii

**Keywords:** cutaneous TB, cutaneous tuberculosis, immune reconstitution inflammatory syndrome, IRIS, scrofuloderma, TB, TB-IRIS, tuberculosis

## Introduction

Tuberculosis (TB) remains a worldwide public health concern and is the second cause of infectious deaths after COVID-19.^1^ Developing countries have a disproportionate burden of disease. Cutaneous TB is a rare manifestation of infection with *Mycobacterium tuberculosis*^2^ and comprises about 1.5% of all extrapulmonary TB cases.^3^

Herein we describe the case of an HIV-negative 77-year-old woman who presented with scrofuloderma and miliary TB, and who later developed immune reconstitution inflammatory syndrome (IRIS) as a response to treatment with rifampin, isoniazid, pyrazinamide, and ethambutol (RIPE).

## Case report

**Fig 1:**
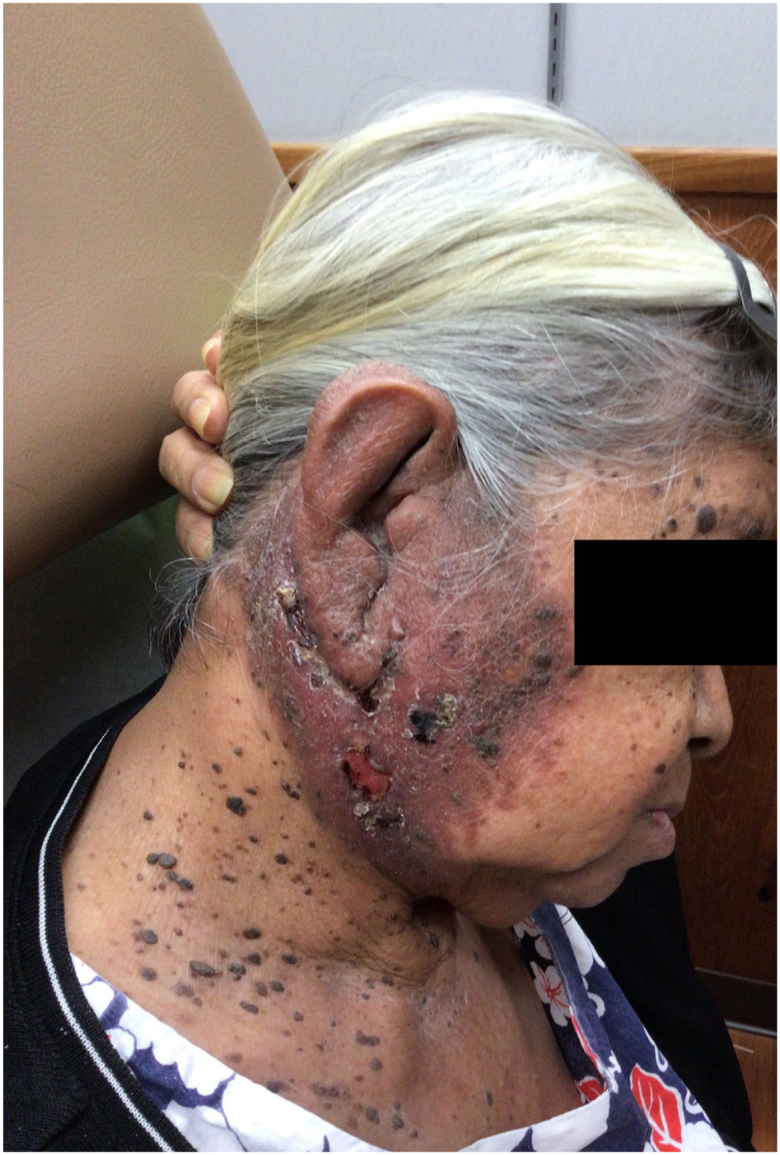
Initial presentation of extensive erythema and hypertrophy.

A 77-year-old Filipina presented to the dermatology clinic with a 1-year history of progressive erythema over the entire right ear and cheek. At the time she reported the area felt swollen, red, and intermittently pruritic. Physical exam showed diffuse induration, infiltration, and crusting of the right ear, extending to the right lateral cheek, neck, and postauricular area (Fig 1).

**Fig 2:**
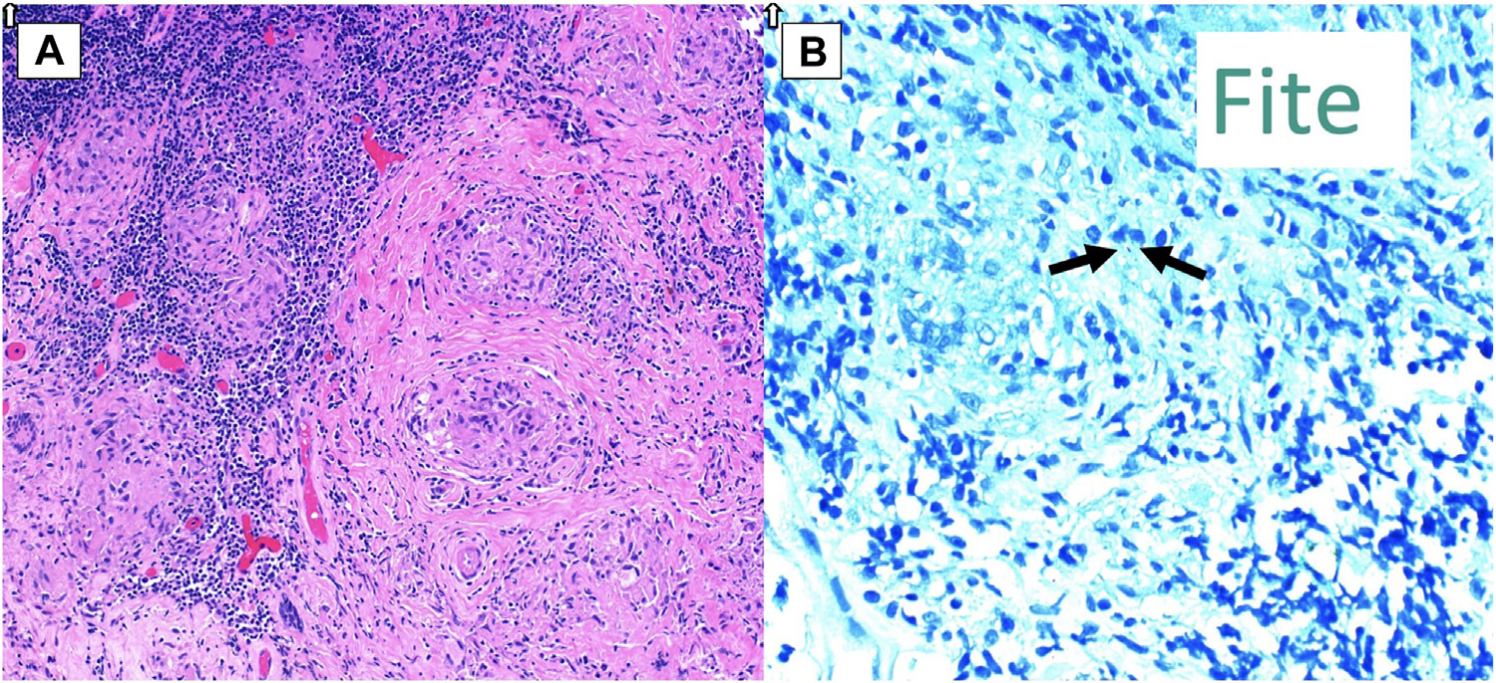
Histopathology of punch biopsy of right helix. **A,** Shows nonnecrotizing epithelioid granulomas with an inflammatory infiltrate (hematoxylin-eosin stain). **B,** Arrows point to rare structure suspicious for acid fast bacilli on Fite stain.

The initial differential diagnosis included severe contact dermatitis, infection, and malignancy. Punch biopsy of the right helix was obtained and showed nonnecrotizing granulomatous infiltrate with epithelioid granulomas and a mixed inflammatory infiltrate, with rare structures suspicious for acid fast bacilli (Fig 2).

QuantiFERON-TB Gold test was negative. Given the acid fast bacilli–like structures on biopsy, tissue cultures were ordered and later grew *M tuberculosis* with sensitivity to RIPE. A DNA probe test was also positive for TB complex. A subsequent chest X-ray showed lung infiltrates consistent with pulmonary TB. A diagnosis of cutaneous TB, specifically scrofuloderma, was made and the patient started on a course of RIPE.

**Fig 3:**
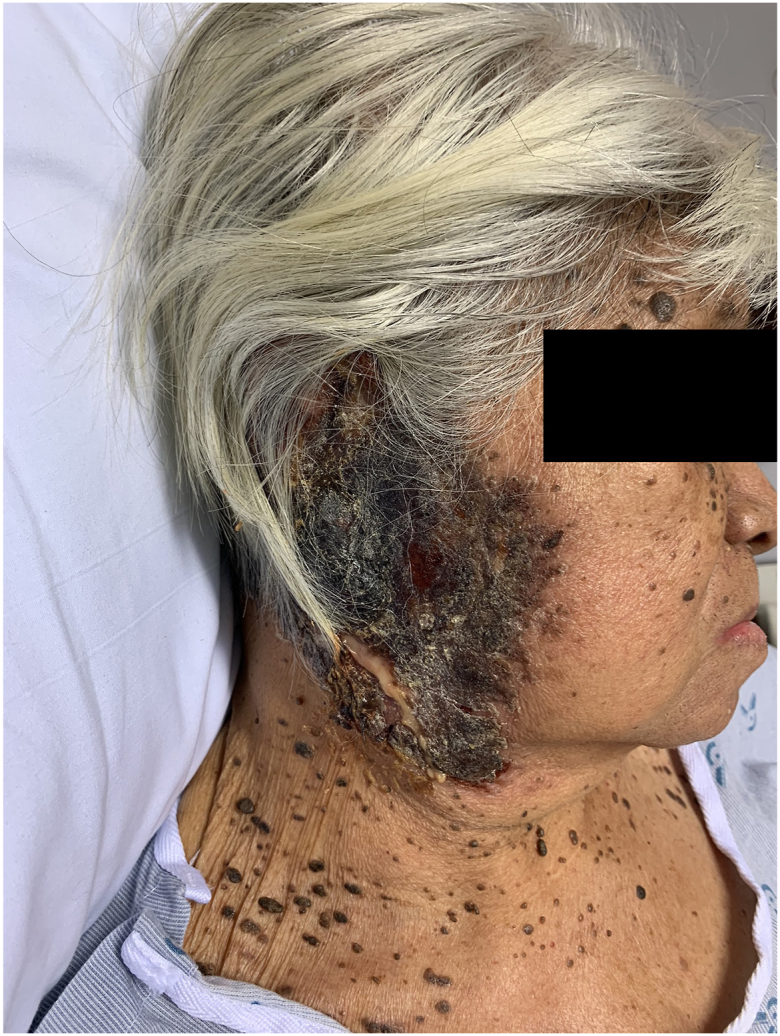
Presentation of immune reconstitution inflammatory syndrome.

One week after RIPE initiation, the patient was hospitalized with worsening pain, crusting, purulent drainage, and necrosis of the affected area (Fig 3).

The patient’s acute worsening following initiation of RIPE was suggestive of IRIS. A superimposed bacterial infection was also suspected, though given the extent of skin damage, it was unlikely her symptoms were purely secondary to an infection. Computed tomography scan of the neck with contrast was done and showed extensive right cervical lymphadenopathy and an irregular enhancing soft tissue mass in the right parotic gland. A computed tomography scan of the chest redemonstrated lung infiltrates, indicative of miliary TB. An HIV test was negative. RIPE was held and the patient was empirically started on ampicillin, sulbactam, and ceftriaxone with prednisone—40 mg daily for 2 weeks, followed by a taper of 20 mg for 2 weeks and 10 mg for 1 week. Wound cultures grew *Pseudomonas aeruginosa* and *Enterobacter cloacae*. Infectious disease was consulted, and the patient transitioned to intravenous vancomycin and meropenem. Prior to discharge, she was transitioned to ceftriaxone. She underwent debridement, irrigation, and skin grafting by otorhinolaryngology. RIPE was eventually restarted without further complications. To date, the patient is recovering well.

## Discussion

Given the growing worldwide prevalence of TB and increasing travel and migration, this case highlights the importance of recognizing less commonly encountered extrapulmonary manifestations of TB, specifically cutaneous TB. The incidence of cutaneous TB in the United States has risen in recent years, with 8300 cases in 2022 compared to 7874 cases reported in 2021.^4^ This trend may be explained by increasing immigration from endemic areas, as well as the burden on public health services by the COVID-19 pandemic.^5^ It is therefore increasingly important to be able to recognize TB in the dermatology practice.

The development of IRIS following therapy with RIPE was also a notable finding in this case, particularly because the patient was HIV-negative. IRIS has been described to develop following initiation of antiretroviral therapy in patients with concurrent HIV and TB infections—TB-IRIS.^6^ In the immunocompromised, TB-IRIS occurs due to sudden reactivation of the immune system by antiretroviral therapy. This corresponds to an increase in CD4+ T-cell count and can lead to a hyperimmune response with widespread damage and potentially necrosis. Although a preventative course of prednisone can be considered for select patients with HIV-associated TB,^7^ this practice is not reinforced in patients without HIV.

In patients without HIV, IRIS has been reported following discontinuation of biologic agents (mainly anti-tumor necrosis factor-alpha), monoclonal antiintegrin antibody, and termination of corticosteroid therapy following stem cell and organ transplants.^7^ Other instances of non-HIV IRIS have been observed in pregnant patients with TB, autoimmune conditions, or malignancies treated with immune checkpoint inhibitors.^7^ However, TB-IRIS in HIV-negative patients without other comorbidities, as in our case, appears to be rare. A similar report of a 55-year-old immunocompetent woman presented with disseminated TB resistant to corticosteroids that was subsequently treated with infliximab.^8^ In HIV-negative patients, TB-IRIS has been proposed to arise due to an amplified cell-mediated immune response against the mycobacteria that are killed by antimycobacterial therapy.^8^ In their review of acutely worsening TB lesions following treatment, Geri et al describe a series of paradoxical reactions, most commonly involving deterioration in the organ of initial presentation.^9^ Other paradoxical reactions were broad –affecting lung, pericardium, pleura, bone, brain, muscle, liver, and ovary—emphasizing the multitude of reactions that can occur in patients without HIV with TB.

Currently, there is no gold standard test to diagnose IRIS. French et al proposed diagnostic criteria for HIV-IRIS, consisting of 2 primary and 3 minor requirements.^10^ Primary criteria require an atypical presentation of opportunistic infection and a decrease in HIV RNA expression by >1 log10 after antiretroviral therapy. The 3 minor criteria include increased CD4+ T-cell count, increased immune response to opportunistic infection, and spontaneous clinical improvement. Given that IRIS is a rare complication of a comparatively rare form of TB (cutaneous), our patient’s clinical course defies the typical presentation of IRIS and, therefore, cannot be evaluated by these diagnostic criteria.

Our report highlights the importance of establishing criteria for diagnosing and managing IRIS arising in HIV-negative patients. Establishing clear guidelines for non-HIV IRIS will guide clinical decision-making in treating the underlying condition. Finally, this case emphasizes the importance of recognizing cutaneous TB in the dermatology setting. Having a high index of suspicion for cutaneous TB, which may mimic other conditions, can allow for a timely diagnosis and improve patient outcomes.

## Conflicts of interest

None disclosed.
